# Supplementary material for: Traveling wave of inflammatory response to regulate the expansion or shrinkage of skin erythema
Source: PLoS One. 2022 Feb 9;17(2):e0263049. doi: 10.1371/journal.pone.0263049 (PMC8827459; doi:10.1371/journal.pone.0263049)
Supplement: S1 Appendix — (DOCX) [file pone.0263049.s001.docx]

## **Supporting Information**

**S1-S3 Fig.**
**Appendix A1-A3.**

## **
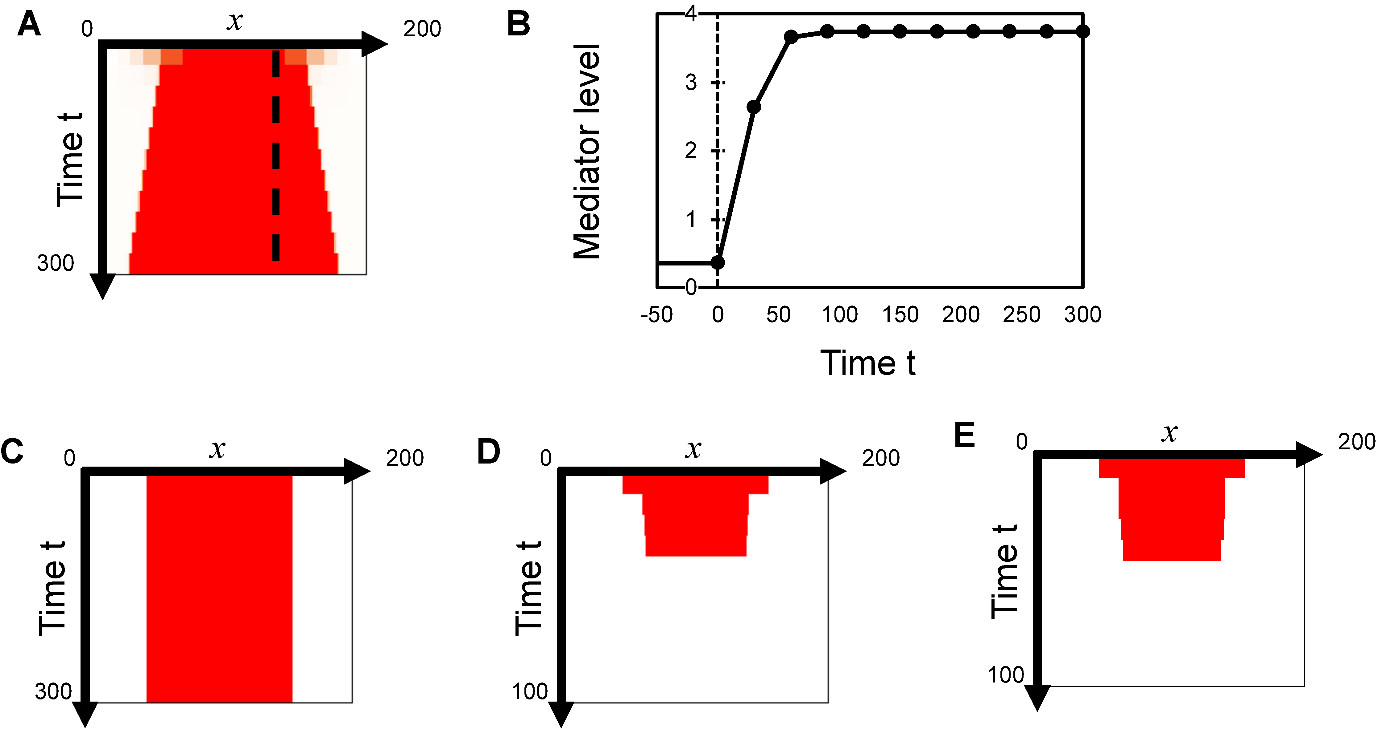
**

**S1 Fig. Diffusion and bistability are necessary for expansion.**

(**A**, **C, D, E**) Spatiotemporal evolution of Eq. (3) in the inflamed area (above the unstable steady state *q* = 0.26; red) at *y*= 100 in a case of the expansion (**A**; *a*=4.0, *b*=0.01, *d*=0.5), and in the absence of the diffusion (**C**; *a*=4.0, *b*=0.01, *d*=0.0), and in the absence of the bistability (**D**; *a*=1.5, *b*=0.01, *d*=0.5, **E**; *a*=1.7, *b*=0.0, *d*=0.5). (**B**) Temporal evolution of mediator levels at *x*= 145 in **A** (dashed line in left panel).

**
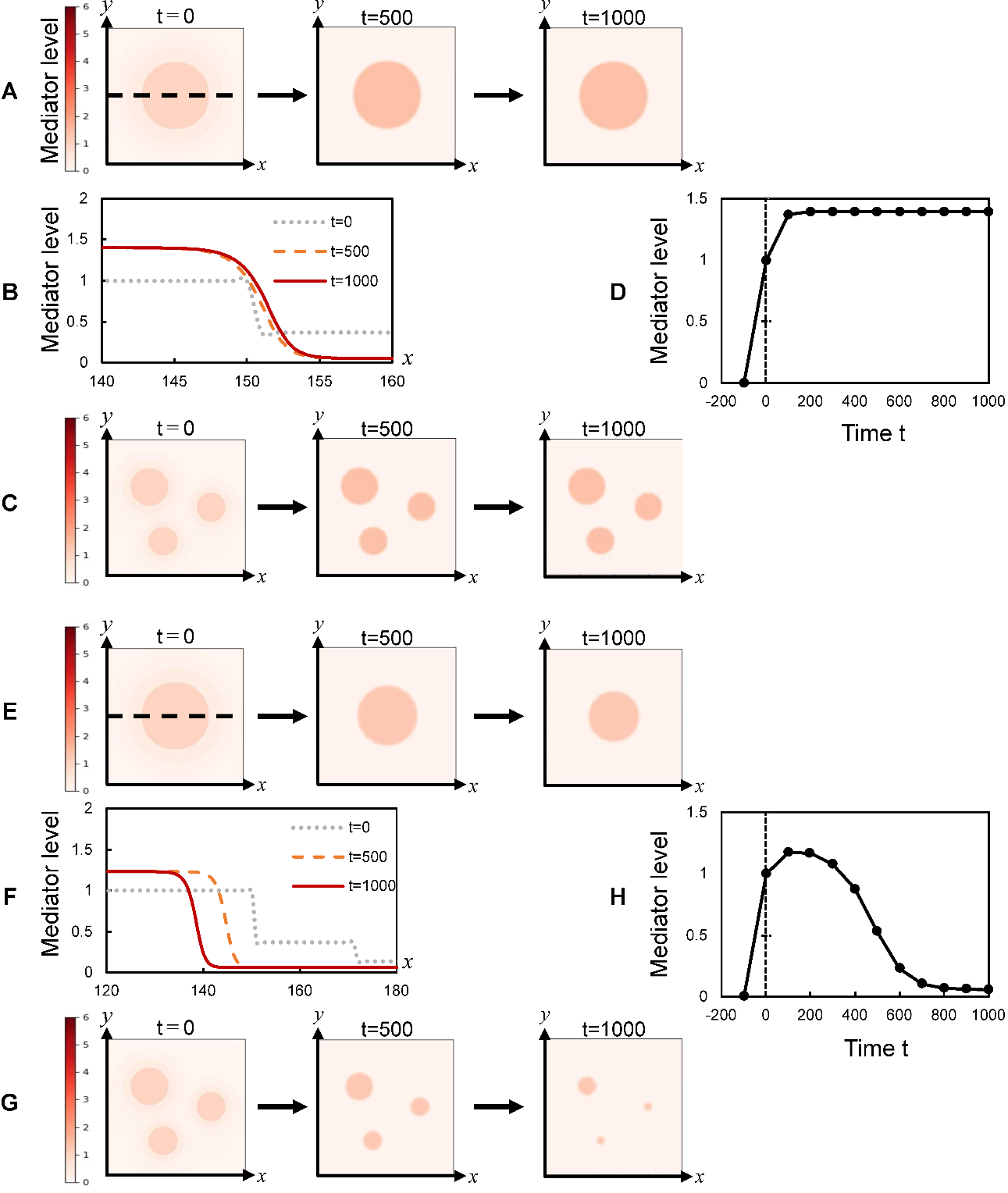
**

**S2 Fig. Slow expansion & shrinkage.**

(**A**, **C, E, G**) Spatiotemporal evolution of inflammatory mediator levels (*q*; inset at the left) upon initial stimulation in a circular area (**A, E**) and in three separate areas (**C, G**). *a*=2.04 in **A, C**. *a*=1.96 in **E, G**. *b*=0.01, *d*=0.5. (**B, F**) Spatial pattern of mediator levels at *y*= 100 in **A** (**B**) and **E** (**F**) (dashed line in left panel). (**D, H**) Temporal evolution of mediator levels at *x*= 145, *y*= 100 in **A (D)** and **E (H)**.


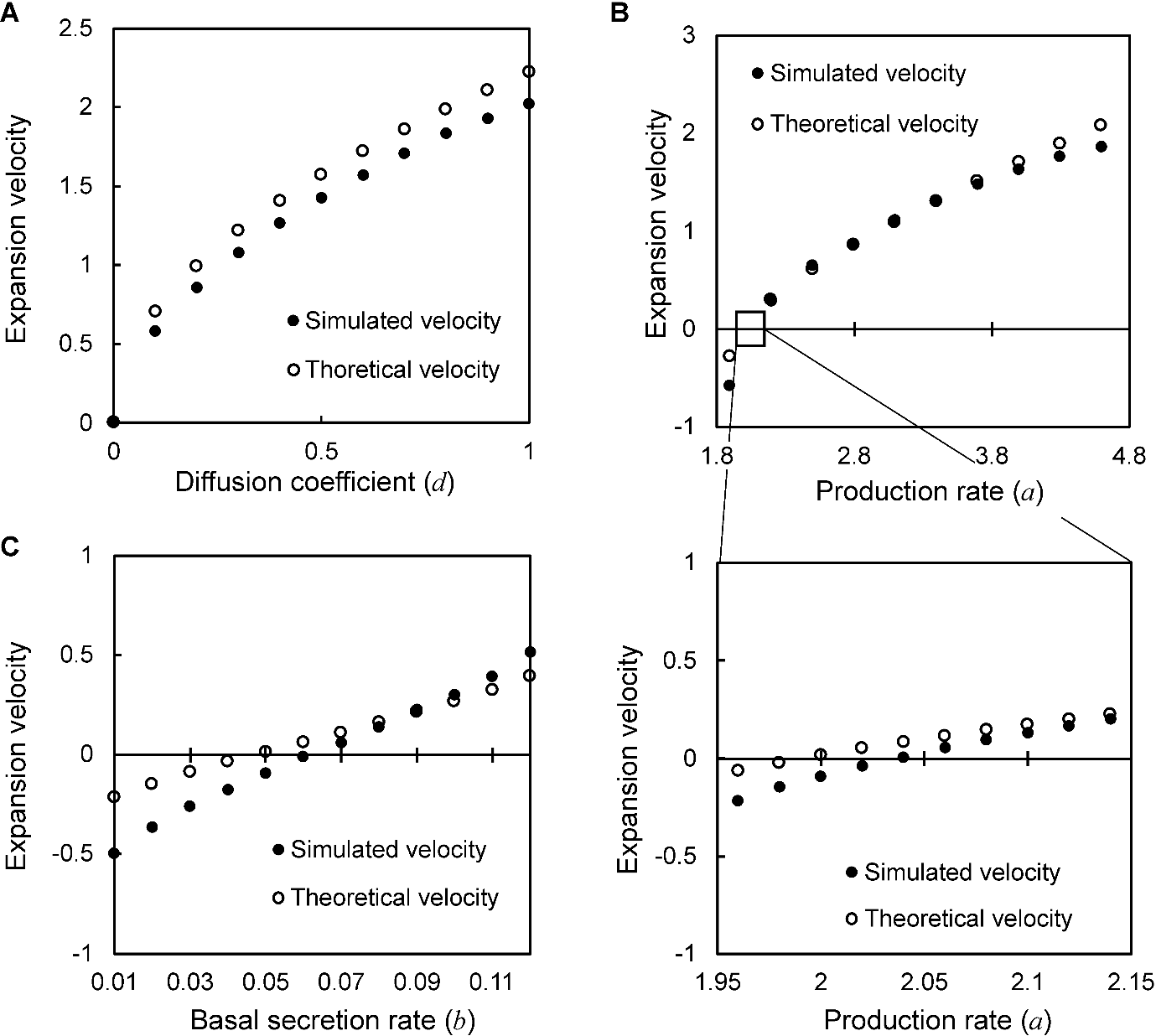


**S3 Fig. Comparison between the simulated velocity (Fig 3) and the theoretically calculated velocity (Eq. 5) with an approximation (Eq. 8).**

(**A**-**C**) Dependence of the expansion velocity on the diffusion coefficient *d* (Eq. 3; **A**), the maximum production rate *a* (**B**), and the basal secretion rate *b* (**C**). Bottom panel of **B** is enlarged view of a segment in the top panel of **B**.

## **Appendix**

### A1. Deriving the normalized model

First, we introduce a normalized time variable as $t\equiv T\gamma$, and Eq. (2) is rewritten as

$\frac{\partial p}{\partial t}=\frac{\alpha p^{n}}{\gamma(p^{n}+{K_{M}}^{n})}+\frac{\beta}{\gamma}-p+\frac{D}{\gamma}\Delta p.$ (S1)

By introducing a normalized concentration variable as $q\equiv\frac{p}{K_{M}},$ Eq. (S1) is rewritten as

$\frac{\partial q}{\partial t}=\frac{\alpha q^{n}}{\gamma K_{M}(q^{n}+1)}+\frac{\beta}{\gamma K_{M}}-q+\frac{D}{\gamma}\Delta q.$ (S2)

By introducing normalized parameters as $a\equiv\frac{\alpha}{\gamma K_{M}}, b\equiv\frac{\beta}{\gamma K_{M}}, d\equiv\frac{D}{\gamma},$Eq. (S2) becomes Eq. (3).

### A2. Relationship between the previous and present model equations

A previous study introduced a reaction–diffusion model of an inflammatory mediator (*p*) - depleted substrate (*s*) system for psoriasis [14]:

$\frac{\partial p}{\partial T}=kp^{2}s+\rho_{p0}-\mu_{p}p+D_{p}\Delta p,$

$\frac{\partial s}{\partial T}=-kp^{2}s+\rho_{s0}-\mu_{s}s+D_{s}\Delta s,$

where *k*, $\rho_{p0}, \mu_{p},D_{p}, \rho_{s0}, \mu_{s},$ and $D_{s}$are constant. Here, we consider a situation where the substrate *s* reaches an equilibrium state ($s=\frac{\rho_{s0}}{kp^{2}+\mu_{s}})$. By substituting this equilibrium concentration of *s* into the above equation for *p*, it becomes

$\frac{\partial p}{\partial T}=\frac{kp^{2}\rho_{s0}}{{kp}^{2}+\mu_{s}}+\rho_{p0}- \mu_{p}p+D_{p}\Delta p.$

This equation becomes identical to Eq. (2) when $, \rho_{s0}=\alpha, \frac{\mu_{s}}{k}={K_{M}}^{n}, \rho_{p0}=\beta, \mu_{p}=\gamma, D_{p}=D, n=2.$

### A3. Analytical derivation of the expansion velocity

A previous mathematical study derived the velocity of the traveling wave in the reaction–diffusion equations [20]. We have applied this theory to Eq. (3), which is rewritten as

$\frac{\partial q}{\partial t}=\frac{1}{q^{2}+1}\left( q-S_{NI} \right)\left( S_{T}-q \right)\left( q-S_{I} \right)+d\Delta q.$

When the denominator of the right side of the equation $q^{2}+1$ is approximated to ${S_{T}}^{2}+1$, this equation becomes

$\frac{\partial q}{\partial t}=A\left( q-S_{NI} \right)\left( S_{T}-q \right)\left( q-S_{I} \right)+d\Delta q,$ (S3)

where $A=\frac{1}{{S_{T}}^{2}+1}$. Eq. (S3) maintains the bistability in the same range of the parameters (*a, b*) as Eq. (3). Suppose that Eq. (S3) has an approximate solution of the wavefront (e.g., Figs. 2A and B), defined as $Q\left( z \right)$:

$Q\left( z \right)\equiv q\left( x,t \right), z=x-vt, Q\left( -\infty\right)=S_{I}, Q\left( \infty\right)=S_{NI},$

where *v* denotes the velocity of the traveling wave*.* Substituting *Q(z)* into Eq. (S3) provides

$d\frac{d^{2}Q}{dz^{2}}+v\frac{dQ}{dz}+A\left( Q-S_{NI} \right)\left( S_{T}-Q \right)\left( Q-S_{I} \right)=0.$ (S4)

As $Q\left( -\infty\right)=S_{I}$ and $Q\left( \infty\right)=S_{NI}$indicate the steady state (i.e., $\frac{dQ}{dz}$=0), $Q$ satisfies

$\frac{dQ}{dz}=B\left( Q-S_{NI} \right)\left( Q-S_{I} \right),$ (S5)

where *B* is a constant. Substituting Eq. (S5) into Eq. (S4), we obtain

$0= \left( Q-S_{NI} \right)\left( Q-S_{I} \right)\{B^{2}d\left( 2Q-S_{NI}-S_{I} \right)+Bv-A(S_{T}-Q)$}

=$\left( Q-S_{NI} \right)\left( Q-S_{I} \right)\left\{ {(2B}^{2}d-A \right)Q-[B^{2}d\left( S_{NI}+S_{I} \right)-Bv-AS_{T}]$}.

Therefore, we must have

$2B^{2}d-A=0, B^{2}d\left( S_{NI}+S_{I} \right)-Bv-AS_{T}=0.$

These two equations provide the velocity Eq. (5).
