# Supplementary material for: Traveling wave of inflammatory response to regulate the expansion or shrinkage of skin erythema
Source: PLoS One. 2022 Feb 9;17(2):e0263049. doi: 10.1371/journal.pone.0263049 (PMC8827459; doi:10.1371/journal.pone.0263049)
Supplement: Attachment — Submitted filename: PONE-D-21-08023_reviewer_annotated.pdf [file pone.0263049.s002.pdf]

PLOS ONE

Traveling wave of inflammatory response to regulate the expansion or shrinking of skin erythema

--Manuscript Draft--

**Manuscript Number:**	PONE-D-21-08023
**Article Type:**	Research Article
**Full Title:**	Traveling wave of inflammatory response to regulate the expansion or shrinking of skin erythema
**Short Title:**	Traveling wave of inflammatory response to regulate the expansion or shrinking of skin erythema
**Corresponding Author:**	Koichi Fujimoto, Ph. D.<br>Osaka University: Osaka Daigaku<br>Toyonaka, Osaka JAPAN
**Keywords:**	skin inflammation; Pattern formation; Reaction-Diffusion; Bistability; Inflammatory mediator; Traveling wave
**Abstract:**	Many skin diseases show a patchy red lesion, called erythema, on the skin. Erythema expands to form a sharply circumscribed lesion upon local transient stimulation. This stimulation causes an inflammatory response, which produces inflammatory mediators in the dermis. The expansion of erythema has been theoretically shown to require the diffusion of mediators; however, other regulations of mediators necessary to control the expansion and shrinkage remain unknown. A candidate of the regulations is positive feedback, which commonly affects mediator production and can generate two distinct stable (i.e., inflamed and non-inflamed) states, referred to as bistability. This bistability causes a state transition between the inflamed and non-inflamed states by transient stimulation. In this study, we theoretically show how diffusion and bistability cause the expansion by using a bistable reaction-diffusion model. Our model showed that diffusion and bistability cause the expansion of the sharply circumscribed lesion via spatially propagating transition from the non-inflamed state to the inflamed state. During expansion, the boundary between the inflamed and non-inflamed areas moves while maintaining a steep gradient. This boundary movement with a constant gradient appears as a traveling wave, which is a general outcome of bistable reaction-diffusion systems. Moreover, by controlling the balance between the inflamed and non-inflamed states, the expansion speed decreases to a negative value, thereby shrinking the inflamed area via the traveling wave . Whether the inflamed area expands or shrinks is determined by the balance between the mediator concentrations at the inflamed and non-inflamed states. The traveling wave of the inflammatory response provides a control principle for erythema expansion and shrinkage, thereby contributing to the development of effective treatments for skin disease.
**Order of Authors:**	Maki Sudo
	Koichi Fujimoto, Ph. D.
**Additional Information:**	
**Question**	**Response**
**Financial Disclosure**<br><br>Enter a financial disclosure statement that describes the sources of funding for the work included in this submission. Review the submission guidelines for detailed requirements. View published research articles from *PLOS ONE* for specific examples.	The funders had no role in study design, data collection and analysis, decision to publish, or preparation of the manuscript.<br><br>Sources of funding?

Powered by Editorial Manager® and ProduXion Manager® from Aries Systems Corporation

This statement is required for submission and **will appear in the published article** if the submission is accepted. Please make sure it is accurate.<br><br>**Unfunded studies**<br>Enter: *The author(s) received no specific funding for this work.*<br><br>**Funded studies**<br>Enter a statement with the following details:<br>• Initials of the authors who received each award<br>• Grant numbers awarded to each author<br>• The full name of each funder<br>• URL of each funder website<br>• Did the sponsors or funders play any role in the study design, data collection and analysis, decision to publish, or preparation of the manuscript?<br>• **NO** - Include this sentence at the end of your statement: *The funders had no role in study design, data collection and analysis, decision to publish, or preparation of the manuscript.*<br>• **YES** - Specify the role(s) played.<br><br>* typeset	
**Competing Interests**<br><br>Use the instructions below to enter a competing interest statement for this submission. On behalf of all authors, disclose any competing interests that could be perceived to bias this work—acknowledging all financial support and any other relevant financial or non-financial competing interests.<br><br>This statement is required for submission and **will appear in the published article** if the submission is accepted. Please make sure it is accurate and that any funding sources listed in your Funding Information later in the submission form are also declared in your Financial Disclosure statement.<br><br>View published research articles from *PLOS ONE* for specific examples.	The authors have declared that no competing interests exist.

**NO authors have competing interests**<br><br>Enter: *The authors have declared that no competing interests exist.*<br><br>**Authors with competing interests**<br><br>Enter competing interest details beginning with this statement:<br><br>*I have read the journal's policy and the authors of this manuscript have the following competing interests: [insert competing interests here]*<br><br>* typeset	
**Ethics Statement**<br><br>Enter an ethics statement for this submission. This statement is required if the study involved:<br><br>• Human participants<br>• Human specimens or tissue<br>• Vertebrate animals or cephalopods<br>• Vertebrate embryos or tissues<br>• Field research<br><br>Write "N/A" if the submission does not require an ethics statement.<br><br>General guidance is provided below. Consult the submission guidelines for detailed instructions. **Make sure that all information entered here is included in the Methods section of the manuscript.**	N/A

**Format for specific study types**<br><br>**Human Subject Research (involving human participants and/or tissue)**<br>• Give the name of the institutional review board or ethics committee that approved the study<br>• Include the approval number and/or a statement indicating approval of this research<br>• Indicate the form of consent obtained (written/oral) or the reason that consent was not obtained (e.g. the data were analyzed anonymously)<br><br>**Animal Research (involving vertebrate animals, embryos or tissues)**<br>• Provide the name of the Institutional Animal Care and Use Committee (IACUC) or other relevant ethics board that reviewed the study protocol, and indicate whether they approved this research or granted a formal waiver of ethical approval<br>• Include an approval number if one was obtained<br>• If the study involved *non-human primates*, add *additional details* about animal welfare and steps taken to ameliorate suffering<br>• If anesthesia, euthanasia, or any kind of animal sacrifice is part of the study, include briefly which substances and/or methods were applied<br><br>**Field Research**<br><br>Include the following details if this study involves the collection of plant, animal, or other materials from a natural setting:<br>• Field permit number<br>• Name of the institution or relevant body that granted permission	
**Data Availability**<br><br>Authors are required to make all data underlying the findings described fully available, without restriction, and from the time of publication. PLOS allows rare exceptions to address legal and ethical concerns. See the PLOS Data Policy and FAQ for detailed information.	Yes - all data are fully available without restriction

A Data Availability Statement describing where the data can be found is required at submission. Your answers to this question constitute the Data Availability Statement and **will be published in the article**, if accepted.<br><br>**Important:** Stating 'data available on request from the author' is not sufficient. If your data are only available upon request, select 'No' for the first question and explain your exceptional situation in the text box.<br><br>Do the authors confirm that all data underlying the findings described in their manuscript are fully available without restriction?	
**Describe where the data may be found in full sentences. If you are copying our sample text, replace any instances of XXX with the appropriate details.**<br><br>• If the data are **held or will be held in a public repository**, include URLs, accession numbers or DOIs. If this information will only be available after acceptance, indicate this by ticking the box below. For example: *All XXX files are available from the XXX database (accession number(s) XXX, XXX.).*<br>• If the data are all contained **within the manuscript and/or Supporting Information files**, enter the following: *All relevant data are within the manuscript and its Supporting Information files.*<br>• If neither of these applies but you are able to provide **details of access elsewhere**, with or without limitations, please do so. For example:<br><br>*Data cannot be shared publicly because of [XXX]. Data are available from the XXX Institutional Data Access / Ethics Committee (contact via XXX) for researchers who meet the criteria for access to confidential data.*<br><br>*The data underlying the results presented in the study are available from (include the name of the third party*	All relevant data are within the manuscript and its Supporting Information files.

and contact information or URL).<br>• This text is appropriate if the data are owned by a third party and authors do not have permission to share the data.<br><br>* typeset	
Additional data availability information:	

Manuscript

Click here to access/download;Manuscript;SudoPaper_20210311.pdf

Traveling wave of inflammatory response to regulate the expansion or shrinking of skin erythema

Maki Sudo, Koichi Fujimoto*

Department of Biological Sciences, Graduate School of Science, Osaka University, Machikaneyama-cho, Toyonaka, 560-0043, Japan

*fujimoto@bio.sci.osaka-u.ac.jp

Keywords

Skin inflammation, Pattern formation, Reaction-diffusion, Bistability, Inflammatory mediator, Traveling wave

Abstract

Many skin diseases show a patchy red lesion, called erythema, on the skin. Erythema expands to form a sharply circumscribed lesion upon local transient stimulation. This stimulation causes an inflammatory response, which produces inflammatory mediators in the dermis. The expansion of erythema has been theoretically shown to require the diffusion of mediators; however, other regulations of mediators necessary to control the expansion and shrinkage remain unknown. A candidate of the regulations is positive feedback, which commonly affects mediator production and can generate two distinct stable (i.e., inflamed and non-inflamed) states, referred to as bistability. This bistability causes a state transition between the inflamed and non-inflamed states by transient stimulation. In this study, we theoretically show how diffusion and bistability cause the expansion by using a bistable reaction-diffusion model. Our model showed that diffusion and bistability cause the expansion of the sharply circumscribed lesion via spatially propagating transition from the non-inflamed state to the inflamed state. During expansion, the boundary between the inflamed and non-inflamed areas moves while maintaining a steep gradient. This boundary movement with a constant gradient appears as a traveling wave, which is a general outcome of bistable reaction-diffusion systems. Moreover, by controlling the balance between the inflamed and non-inflamed states, the expansion speed decreases to a negative value, thereby shrinking the inflamed area via the traveling wave. Whether the inflamed area expands or shrinks is determined by the balance between the mediator concentrations at the inflamed and non-inflamed states. The traveling wave of the inflammatory response provides a control principle for erythema expansion and shrinkage, thereby contributing to the development of effective treatments for skin disease.

Not shown to require. A model has shown that diffusible mediators could explain observed propagation. That doesn't show that such mediators are required.

Maybe "modulation" is more appropriate than "regulation"?

Steepness depends on parameters?

What does "the balance" mean? Expansion or shrinkage depends on a well known integral condition and not only the steady state values but also the nonlinearity between them.

?

ors ?

for

can be

Such as ?

Background

Numerous inflammatory skin diseases, including eczema, urticaria, psoriasis, infectious diseases, and lymphomas, lead to the formation of patchy red lesion areas on the skin called erythema [1–5]. Erythema is caused by various pathogenic factors, such as physical stimulations, chemical drugs, and bacterial infections (Fig 1A. i) [1]. These factors lead to an inflammatory response, that is, secretion of inflammatory mediators, such as cytokines (e.g., TNF-α and IL-1β) and histamine (Fig 1A. ii) [6–10]. These mediators increase the blood volume in the dermal blood vessels (Fig 1A. iii) [9,10]. This increase in the blood volume appears as erythema (Fig 1A. iv). This pathomechanism of erythema is common in various skin diseases. Another common phenomenon of erythema is expansion. The erythema centrifugally expands from only local transient stimulation ~~site~~ to form a sharply circumscribed lesion (Fig 1B) [1, 4]. Expansion of multiple erythemas leads to their fusion [1]. This autonomous expansion is an indispensable event during disease progression. In this kind of pathological case, the inflammatory response persists and the concentrations of mediators fail to return to the original levels, whereas a normal inflammatory response initiates a temporal increase in the level of mediators, which return to the original levels [11–14]. Controlling the inflammatory response to suppress the expansion speed and further shrink the erythema can offer indications for developing effective treatments. Nevertheless, how the expansion and shrinkage of the erythema are controlled by the inflammatory response remains unclear, because of the experimental difficulty in detecting spatiotemporal evolution of the inflammatory response in the skin dermis.

Why so difficult? Redness should be easy to observe.

Mathematical modeling of such skin pathogenesis and their computer simulations have produced valuable predictions in recent years [15–18]. For spatiotemporal evolution, the inflammatory response has been mathematically modeled to demonstrate the expansion of erythema. One model incorporated experimentally known biochemical or transcriptional regulation of cytokines and their intercellular diffusion and highlighted the expansion of the sharply circumscribed lesion [4]. This type of model incorporates the reaction and diffusion of molecules and is called a reaction-diffusion model [19,20]. Another reaction-diffusion model incorporating self-activation, that is, positive feedback, and

Only “showed” in the context of the assumptions made in those models. This does not show that biologically mediators are required (other mechanisms are not ruled out).

inhibition of histamine has also shown expansion [3]. These models showed that diffusion of mediators is required for erythema expansion. Furthermore, the common regulation of mediators responsible for the expansion in these two models is the positive feedback that can generate two distinct, stable states (i.e., an inflamed and a non-inflamed state), namely, bistability. This bistability causes a persistent transition from the non-inflamed state to the inflamed state by transient stimulation [12]. However, the mechanism of how diffusion and bistability cause expansion and whether they can further lead to the shrinkage of erythema remain unknown.

Biologically or mathematically? It is well known mathematically that bistability can lead to forward or reverse waves.

In this study, we aim to develop a bistable reaction-diffusion model to determine whether and how diffusion and bistability cause expansion and shrinkage. The expansion of a sharply circumscribed lesion appears as a traveling wave, that is, spatial propagation of the transition from the non-inflamed state to the inflamed state. We also demonstrate that diffusion and bistability are necessary, but not sufficient, to cause expansion; that is, they can also shrink the erythema. We clarified the detailed conditions of bistability required to expand or shrink erythema.

why necessary?

need to give context - there is lots of published relevant mathematical modelling

Methods

To analyze the expansion of erythema, we formulated a reaction-diffusion model [19,20]. We considered a two-dimensional space along the skin surface, assuming a homogeneous distribution of mediator-secreting cells, such as immune cells and keratinocytes. We first formulated the following equation by introducing the observed biochemical or transcriptional regulation of the inflammatory mediator’s concentration (p) in the intracellular and extracellular environments into an ordinary differential equation (ODE):

$$\frac{dp}{dt} = \frac{\alpha p^n}{p^n + K_m} + \beta - \gamma p \quad . \qquad (1)$$

usually K_m^n so p, K_m have same units

The first, second, and third terms represent the induction of own production (positive feedback) [21], basal secretion [22], and degradation [23], respectively. Here, α, n, K_m, β, and γ denote the maximum rate of production, Hill constant, threshold of production, basal secretion rate, and degradation rate,

respectively. The production rate (α) is experimentally suggested to increase with the deterioration of the skin microbiome [24]. The basal secretion rate (β) increases with a deficiency of the skin barrier structure [22]. Therefore, α and β are parameters that represent skin conditions.

Next, in order to incorporate the bistability, that is, two stable state, we set the value of the parameters (α = 1.0, n = 2, K_m = 0.55, β = 0.039, and γ = 0.68, unless specified in the text or figure captions). These two stable states are given by low and high concentrations, corresponding to the non-inflamed state and the inflamed lesion state (S_{NI} and S_I, respectively, in Fig 1C), respectively. In this setting, the model has one unstable steady state, which corresponds to a threshold concentration (S_T in Fig 1C) for the transition between the two stable states. The S_{NI} is maintained for a subthreshold perturbation (i.e., below the concentration of S_T), whereas it transits to the S_I for a suprathreshold one (i.e., above the concentration of S_T). The S_I also transits to S_{NI} when the concentration decreases to below the concentration of S_T.

Then, we introduced the diffusion to formulate a reaction-diffusion equation.

$$\frac{\partial p}{\partial t} = \frac{\alpha p^n}{p^n + K_m} + \beta - \gamma p + D\Delta p \quad , \qquad (2)$$

where D and Δ in the fourth term denote the diffusion coefficient and the Laplacian operator ($\frac{\partial^2}{\partial x^2}+\frac{\partial^2}{\partial y^2}$), respectively [25]. D = 0.5, unless specified in the text or figure captions.

Finally, we set up an initial condition for the external pathogenic factors leading to the secretion of large amounts of mediators. This initial suprathreshold perturbation to the skin causes a transition from the non-inflamed state to the inflamed state and causes a small inflamed lesion of approximately a few millimeters [1,9]. Thus, the initial conditions of the model simulation (Eq. 2) are set as a small circular inflamed area of a high concentration of p with a radius of approximately a few centimeters, which is given by the two-dimensional Gaussian distribution. Given this initial condition, the model was simulated using the Euler method in two-dimensional geometries under the periodic boundary condition. The value of dt = 0.1 and the numerical stability of the numerical solution is validated.

In time? Insufficient detail. How are the diffusion terms approximated?

Fig 1. Modeling of the expansion of erythema.

(A) Process of the inflammatory response for erythema development. When keratinocytes in the epidermis and resident immune cells in the dermis are stimulated (i), they secrete inflammatory mediators that induce their own production from these mediator-secreting cells (ii). The mediators diffuse in the dermis and cause the dilation of local blood vessels (iii). The dilation appears as redness on the skin surface and erythema develops (iv).

(B) A two-dimensional illustration of erythema expansion showing sharply circumscribed boundaries and fusion.

(C) Kinetics of bistability in Eq. (1) represented by a production rate of mediators (*dp/dt*) as a function of the concentration (p). Two filled circles represent stable steady states (S_{NI}, S_I), whereas the hollow circle indicates an unstable steady state (S_T). This system can switch between the states of stable low (S_{NI}) and high (S_I) concentration states, depending on perturbation such as initial stimulation or diffused mediators ($\alpha = 1.0$).

Results

Diffusion and bistability necessary for expansion

Using this bistable reaction-diffusion model of inflammatory mediators, we examined whether diffusion and bistability are necessary for the expansion of the sharply circumscribed lesion. The model simulations consistently showed that an inflamed area was initially caused by a transient perturbation onto a small area and subsequently expanded centrifugally over time (Fig 2A). During expansion, the inflamed area showed a clear boundary where the mediator concentration exhibited a steep gradient in space (Fig 2B), accounting for the sharply circumscribed lesion observed during erythema expansion (Fig 2C). This model further reproduced the fusion of multiple inflamed areas

depends on parameters

(Fig 2D). We then examined whether diffusion is necessary for this expansion. When the diffusion coefficient (D) was small, an inflamed area was initially observed; however, this area did not expand and remained constant over time (Fig 2E). We next examined whether bistability is necessary for expansion. When bistability was lost by decreasing the production rate (α), a local transient stimulation caused an inflamed area, but the area did not expand (Fig 2F). Thus, these results confirmed that diffusion and bistability are necessary for the expansion of sharply circumscribed lesions.

Expansion caused by a traveling wave of the transition from a non-inflamed to inflamed state

This expansion follows typical spatiotemporal changes in inflammatory mediator concentration. First, an initial suprathreshold perturbation locally induced an inflamed area, producing many mediators by positive feedback (Fig 2A, B, time = 0). These mediators diffused to the adjacent non-inflamed area, and subsequently became a suprathreshold perturbation, causing the transition from the non-inflamed state (S_{NI}) to the inflamed state (S_I). This state transition occurs at the boundary between the inflamed area and the non-inflamed area (Fig 2A, B, time = 500). This series of events, that is, positive feedback of production, diffusion, and state transition in the adjacent area, is autonomously propagated to the surrounding non-inflamed area, resulting in the expansion of the inflamed area (Fig 2A, B). During expansion, the boundary between the inflamed and non-inflamed areas moved while maintaining a steep gradient (Fig 2B) because the mediators are produced, degraded, and diffused at constant rates in the inflamed area. This boundary movement with a constant gradient appears as a traveling wave, which is the general outcome of bistable reaction-diffusion systems [19]. Moreover, the diameter of the inflamed area increased at a constant rate over time (Fig 2D), showing a constant velocity of the traveling wave. Therefore, diffusion and bistability caused the expansion of the sharply circumscribed lesion via the traveling wave of transition from the non-inflamed to the inflamed state.

Fig 2. Diffusion and bistability are necessary for expansion via the traveling wave.

(A, D) Spatiotemporal evolution of inflammatory mediator levels (p; inset at the top) upon initial stimulation in a circular area (**A**) and in three separated areas (**D**; top panel).

(B) Spatial pattern of mediator levels at $y = 100$ in **A** (dashed line in top panel). The boundary between the inflamed and non-inflamed areas temporally moves with a steep gradient.

(C, E, F) Spatiotemporal changes in the inflamed area (above the unstable steady state $p = 0.45$; red) at $y = 100$ in **A** (**C**), in the absence of the diffusion (**E**; $D = 0$), and in the absence of the bistability (**F**; $\alpha = 0.9$).

No, qualitatively different. D changes speed but not sign, other parameters can change the sign.

Control of bistability or diffusion to suppress the expansion speed

We examined whether the expansion speed of the inflamed area can be suppressed by controlling diffusion and bistability. The expansion speed monotonically decreased with decreasing diffusion coefficient (D) (Fig 3A). Manipulation of the parameters controlling the positive feedback activity (i.e., α, β, and γ) showed a similar effect: the expansion speed decreased with decreasing the production rate (α) or the basal secretion rate (β), or increasing the degradation rate (γ) (Fig 3B-F and S1 Fig). As α or β decreases or γ increases, the expansion speed continuously decreases and falls below behind zero at a threshold value of α, β, and γ (Fig 3B, C, and D). Thus, these results showed that the expansion speed was suppressed by diffusion and positive feedback activity for a given bistability.

This is well known. Zero speed when integral of the temporal dynamics is zero.

Erythema shrinkage by controlling bistability

Below the threshold value of α or β or above the threshold of γ, the expansion speed became negative, resulting in the shrinkage of the inflamed area via the traveling wave (Fig 3B-D, G, and S2 Fig). However, this shrinkage never occurred when D decreased (Fig 3A). Therefore, the bistable reaction-diffusion model showed that erythema could be shrunk by decreasing positive feedback activity for a given bistability.

---

from Murray:

which determine a and the unique wavespeed c as

$$a = \left(\frac{A}{2D}\right)^{1/2}, \quad c = \left(\frac{AD}{2}\right)^{1/2}(u_1 - 2u_2 + u_3). \tag{13.87}$$

Fig 3. Expansion speed controlled by diffusion and positive feedback for given bistability. **(A-D)** The dependence of expansion speed on the diffusion coefficient (D; **A**), the production rate (α; **B**), the basal secretion rate (β; **C**), and the degradation rate (γ; **D**). **(E-G)** Spatiotemporal changes of the inflamed area (above the unstable steady state p = 0.37; **E**, 0.42; **F**, 0.5; **G**, respectively) for three different values of production rate (E, F, G in **B**).

Balance between the inflamed and non-inflamed state concentrations determines expansion or shrinkage

Explain the connection to the model and that this is an approximation.

variable; not a control par

Finally, we clarify the role of mediator concentration on expansion, shrinkage, and the speed at which they occur. Mathematically, the expansion speed is represented by the diffusion coefficient (D) and the concentration of three steady states (i.e., S_{NI}, S_T, S_I), which pass through the inflammatory responses:

"determined" ⟷ "approximated"?

at the

$$c = \left(\frac{AD}{2}\right)^{1/2}(S_{NI} + S_I - 2S_T), \tag{3}$$

from Murray text

where A is a positive constant in a simplified reaction-diffusion equation (See Appendix) [19]. This speed is proportional to the square root of the diffusion coefficient (D; Fig 3A), and more interestingly, to the difference between the concentration of stable (S_{NI} and S_I) and unstable (S_T) states. The calculated speed agreed with the simulated expansion speed in the present model (Eq. 2) over a wide range of model parameters (i.e., D, α, β, and γ; S3 Fig A-D). When S_T is closer to S_{NI} than to S_I, the speed is positive, indicating the inflamed area expansion (blue lines in Fig 4). As S_{NI} and S_I are smaller or S_T is larger, according to decreasing the production rate (α; Fig 4A) or the basal secretion rate (β; Fig 4B), or to increasing the degradation rate (γ; Fig 4C), the expansion speed is suppressed toward zero at an equal distance from S_T to S_{NI} and to S_I (black lines in Fig 4). The speed is negative, indicating the shrinkage of the inflamed area when S_T becomes closer to S_I than to S_{NI} (red lines in Fig 4). Therefore, depending on whether threshold concentration is closer to non-inflamed or inflamed, erythema expands or shrinks, respectively. This balance of mediator concentrations (i.e., how close

not really

not equal here

the threshold is to the non-inflamed or inflamed state), further determines speed of expansion or shrinkage (Fig 5).

Fig 4. Balance of mediator concentration regulates expansion or shrinkage.
Kinetics of bistability in Eq. (1) represented by the production rate of mediators (*dp/dt*) as a function of the concentration (p). Two filled circles (S_{NI}, S_I) represent stable steady states, whereas the hollow circle (S_T) indicates an unstable steady state. (**A**) $\alpha = 1.09, 1.03, 0.97$. (**B**) $\beta = 0.045, 0.039, 0.03$. (**C**) $\gamma = 0.65, 0.68, 0.72$. Blue and red lines represent models for erythema expansion and shrinkage, respectively, while the black line applies to zero expansion speed.

Fig 5. Traveling wave of inflammatory response regulates the expansion or shrinkage of erythema.

Discussion

Diffusion and bistability cause both expansion and shrinkage of erythema as the traveling wave

evidence - how crucial, what's normal, abnormal ...

Erythema expansion is a crucial process in disease progression. Recent theoretical studies have shown two independent mechanisms of how the pathological inflammatory response causes expansion [4,6]. One is the diffusion of inflammatory mediators, whereas the other is the positive feedback, which can generate bistability. However, the mechanism of how diffusion and bistability can cause expansion remains unknown. Furthermore, the underlying mechanism of the shrinkage of erythema in the normal inflammatory response is not identified. In this paper, we theoretically show that diffusion and bistability synergistically cause both expansion and shrinkage as the traveling wave of the

Don't think ref [6] is theoretical.

inflammatory response (Figs 2 and 3). Moreover, whether the inflamed area expands or shrinks is determined by the balance between the non-inflamed (S_{NI}) and inflamed state (S_I) concentrations, relative to the threshold state (S_T, Figs 4 and 5). This balance is controlled by the observed biochemical or transcriptional regulation of the inflammatory response, including positive feedback, basal secretion, and degradation (Figs 3 and 4). This balance of bistable states summarizes a new control principle for the normal and pathological inflammatory responses.

good

Potential application of treatments

This theoretical principle on the expansion speed reduction and even shrinkage of the inflamed area (Figs 3 and 5) provides possible treatment methods to reduce the production rate (α) or the basal secretion rate (β). The production rate (α) is known to increase owing to the deterioration of the skin microbiome [11,24]. Thus, some treatments that improve the composition of the skin microbiome will lead to suppression of expansion and shrinkage of erythema. In addition, as the basal secretion rate (β) is known to increase owing to the deficiency of a skin barrier protein [11,22], some treatments to increase this protein activity will lead to suppression of expansion and shrinkage. Thus, the principle predicts that the expansion speed is controlled by the positive feedback activity depending on skin conditions, such as the skin barrier and microbiome. This prediction will be experimentally validated by measuring the expansion speed under different skin conditions. Further, this measurement will allow the principle to quantitatively examine how expansion and shrinkage are controlled by the inflammatory response and to offer further insights useful for developing more effective treatments.

other regulators - not only barriers (microbiome-cytokines?

Limitation of the present model

Erythema expansion is clinically observed to stop at a certain size [3]. This phenomenon may be controlled by anti-inflammatory mediators, such as IL-10 and TGF-β [8,9]. Future studies should incorporate this type of mediator into the present model and examine how anti-inflammatory and inflammatory mediators synergistically control the expansion of erythema.

There is lots of published modelling of pro- and anti-inflammatory signalling and bistability, some of which should be discussed in this context.

Conclusions

In this paper, we demonstrated that diffusion and bistability are necessary to cause expansion and shrinkage as a traveling wave. The positive feedback activity of mediator production regulates the transition between the non-inflamed and inflamed states, thereby determining whether the inflamed area expands or shrinks (Fig 5). Moreover, regulation of the traveling wave of the inflammatory response under the balance between non-inflamed and inflamed states provides a control principle for the spatiotemporal evolution of erythema, which can help to develop effective treatments.

Appendix

Analytical derivation of the expansion speed

The bistable nature (Fig 1C) maintained even when Eq. (2) is simplified to

$$\frac{\partial p}{\partial t} = A(p - S_{NI})(S_T - p)(p - S_I) \ + D\Delta p \ , \qquad (4)$$

under an assumption that $\frac{\gamma}{{S_{NI}}^n + K_m}$ is approximated to a constant A [19]. Suppose that Eq. (4) has wavefront solutions of the form (e.g., Figs 2A and B) defined as $P(z)$:

$$P(z) \equiv p(x,t), z = x - ct, P(-\infty) = S_I,\ P(\infty) = S_{NI},$$

where c denotes the wavespeed. Substituting $P(z)$ into Eq. (4) provides

$$D\frac{d^2P}{dz^2} + c\frac{dP}{dz} + A(P - S_{NI})(S_T - P)(P - S_I) = 0. \qquad (5)$$

As $P(-\infty)=S_I$ and $P(\infty)=S_{NI}$ indicate the steady state (i.e., $dP/dz=0$), P satisfies

$$\frac{dP}{dz} \ = \ a(P - S_{NI})(P - S_I) \ \ , \qquad (6)$$

where a is a constant. Substituting Eq. (6) into Eq. (5), we obtain

$$0 \ = (P - S_{NI})(P - S_I) \ \ \{a^2D(2P - S_{NI} - S_I) + ac - A(S_T - P)\}$$

$$= (P - S_{NI})(P - S_I) \ \ \{(2a^2D - A)P - [a^2D(S_{NI}+S_I) - ac - AS_T]\}.$$

Therefore, we must have

$$2a^2D - A = 0, a^2D(S_{NI}+S_I) - ac - AS_T = 0.$$

These two equations provide wavespeed c in Eq. (3).

This calculation is in large part directly from JD Murray's book "Mathematical Biology".

Notes exploring the link between this and other models.

$$\frac{\partial a}{\partial t} = a^2 s - (f + c)a + D_a \nabla^2 a$$
$$\frac{\partial s}{\partial t} = -a^2 s + (1 - s)f + D_s \nabla^2 s$$

to and Miorandi, 2010; Yamamoto et al., 201

$k = 1, \ \rho_{a0} = 0, \ \mu_a = f + c, \ \rho_{s0} = f, \ \mu_s = f.$

Acknowledgments

We thank M. S. Kitazawa and K. Matsushita (Osaka Univ., Japan) for stimulating discussion.

$$\frac{\partial a}{\partial t} = ka^2 s + \rho_{a0} - \mu_a a + D_a \nabla^2 a,$$
$$\frac{\partial s}{\partial t} = -ka^2 s + \rho_{s0} - \mu_s s + D_s \nabla^2 s.$$

$\dot{a}=0: s = \frac{\mu_a a - \rho_{a0}}{ka^2}$

$\dot{s}=0: s = \frac{\rho_{s0}}{ka^2+\mu_s}$

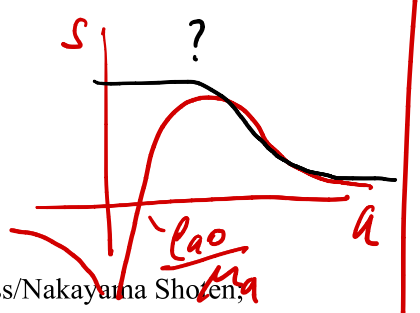

Author Contributions

Conceptualization: MS, KF.

Methodology: MS.

Investigation: MS.

Formal analysis: MS.

Writing – original draft preparation: MS, KF.

Writing – review and editing: MS, KF.

Supervision: KF.

References

1. Shimizu H. Shimizu's textbook of dermatology. Hokkaido University Press/Nakayama Shoten; 2007. 564 p.

2. Segel LA, Perelson AS, Hyman JM, Klaus SN. Rash theory. Theor Exp Insights Immunol. 1992;333–52.

3. Seirin-Lee S, Yanase Y, Takahagi S, Hide M. A single reaction-diffusion equation for the multifarious eruptions of urticaria. PLoS Comput Biol. 2020;16(1):e1007590.

4. Ringham L, Prusinkiewicz P, Gniadecki R. Skin patterning in psoriasis by spatial interactions between pathogenic cytokines. iScience. 2019;20:546–53.

5. Vig DK, Wolgemuth CW. Spatiotemporal evolution of erythema migrans, the hallmark rash of lyme disease. Biophys J. 2014;106(3):763–8.

6. Zhang J-M, An J. Cytokines, inflammation, and pain. Int Anesthesiol Clin. 2007;45(2):27–37.

7. Fernández TD, Canto G, Blanca M. Molecular mechanisms of maculopapular exanthema: Curr Opin Infect Dis. 2009;22(3):272–8.

8. Nestle FO, Di Meglio P, Qin J-Z, Nickoloff BJ. Skin immune sentinels in health and disease.

special case? $\rho_{a0}=0$ $\dot{a}=0$ $s=\frac{\mu_a}{ka}$ and $a=0$

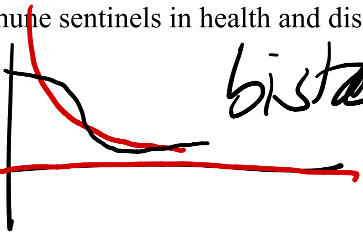

bistable

Nat Rev Immunol. 2009;9(10):679–91.
9. Murphy K, Weaver C. Janeway's immunobiology. 9th ed. New York, NY: Garland Science; 2016. 924 p.
10. Kabashima K, Honda T, Ginhoux F, Egawa G. The immunological anatomy of the skin. Nat Rev Immunol. 2019;19(1):19–30.
11. Dainichi T, Hanakawa S, Kabashima K. Classification of inflammatory skin diseases: A proposal based on the disorders of the three-layered defense systems, barrier, innate immunity and acquired immunity. J Dermatol Sci. 2014;76(2):81–9.
12. Yeo S-Y, Lee K-W, Shin D, An S, Cho K-H, Kim S-H. A positive feedback loop bi-stably activates fibroblasts. Nat Commun. 2018;9(1):3016.
13. Valeyev NV, Hundhausen C, Umezawa Y, Kotov NV, Williams G, Clop A, et al. A systems model for immune cell interactions unravels the mechanism of inflammation in human skin. PLoS Comput Biol. 2010;6(12):e1001024.
14. Domínguez-Hüttinger E, Christodoulides P, Miyauchi K, Irvine AD, Okada-Hatakeyama M, Kubo M, et al. Mathematical modeling of atopic dermatitis reveals "double-switch" mechanisms underlying 4 common disease phenotypes. J Allergy Clin Immunol. 2017;139(6):1861-1872.e7.
15. Gilmore S, Landman KA. Is the skin an excitable medium? pattern formation in erythema gyratum repens. J Theor Med. 2005;6(1):57–65.
16. Denda M, Denda S, Tsutsumi M, Goto M, Kumamoto J, Nakatani M, et al. Frontiers in epidermal barrier homeostasis - an approach to mathematical modelling of epidermal calcium dynamics. Exp Dermatol. 2014;23(2):79–82.
17. Nakaoka S, Kuwahara S, Lee C, Jeon H, Lee J, Takeuchi Y, et al. Chronic inflammation in the epidermis: a mathematical model. Appl Sci. 2016;6(9):252.
18. Hurault G, Domínguez-Hüttinger E, Langan SM, Williams HC, Tanaka RJ. Personalized prediction of daily eczema severity scores using a mechanistic machine learning model. Clin Exp Allergy. 2020;50(11):1258–66.
19. Murray JD. Mathematical biology. 3rd ed. New York: Springer; 2002.
20. Kondo S, Miura T. Reaction-diffusion model as a framework for understanding biological

pattern formation. Science. 2010;329

21. Bonizzi G, Karin M. The two NF-κB activation pathways and their role in innate and adaptive immunity. Trends Immunol. 2004;25(6):280–8.

22. Bäsler K, Brandner JM. Tight junctions in skin inflammation. Pflüg Arch - Eur J Physiol. 2017;469(1):3–14.

23. Zhao W, Oskeritzian CA, Pozez AL, Schwartz LB. Cytokine production by skin-derived mast cells: endogenous proteases are responsible for degradation of cytokines. J Immunol. 2005;175(4):2635–42.

24. Meisel JS, Sfyroera G, Bartow-McKenney C, Gimblet C, Bugayev J, Horwinski J, et al. Commensal microbiota modulate gene expression in the skin. Microbiome. 2018;6(1):20.

25. Cornelissen LH, Bronneberg D, Bader DL, Baaijens FPT, Oomens CWJ. The Transport Profile of Cytokines in Epidermal Equivalents Subjected to Mechanical Loading. Ann Biomed Eng. 2009;37(5):1007–18.

Figure 1 Click here to access/download;Figure;Fig1.tif

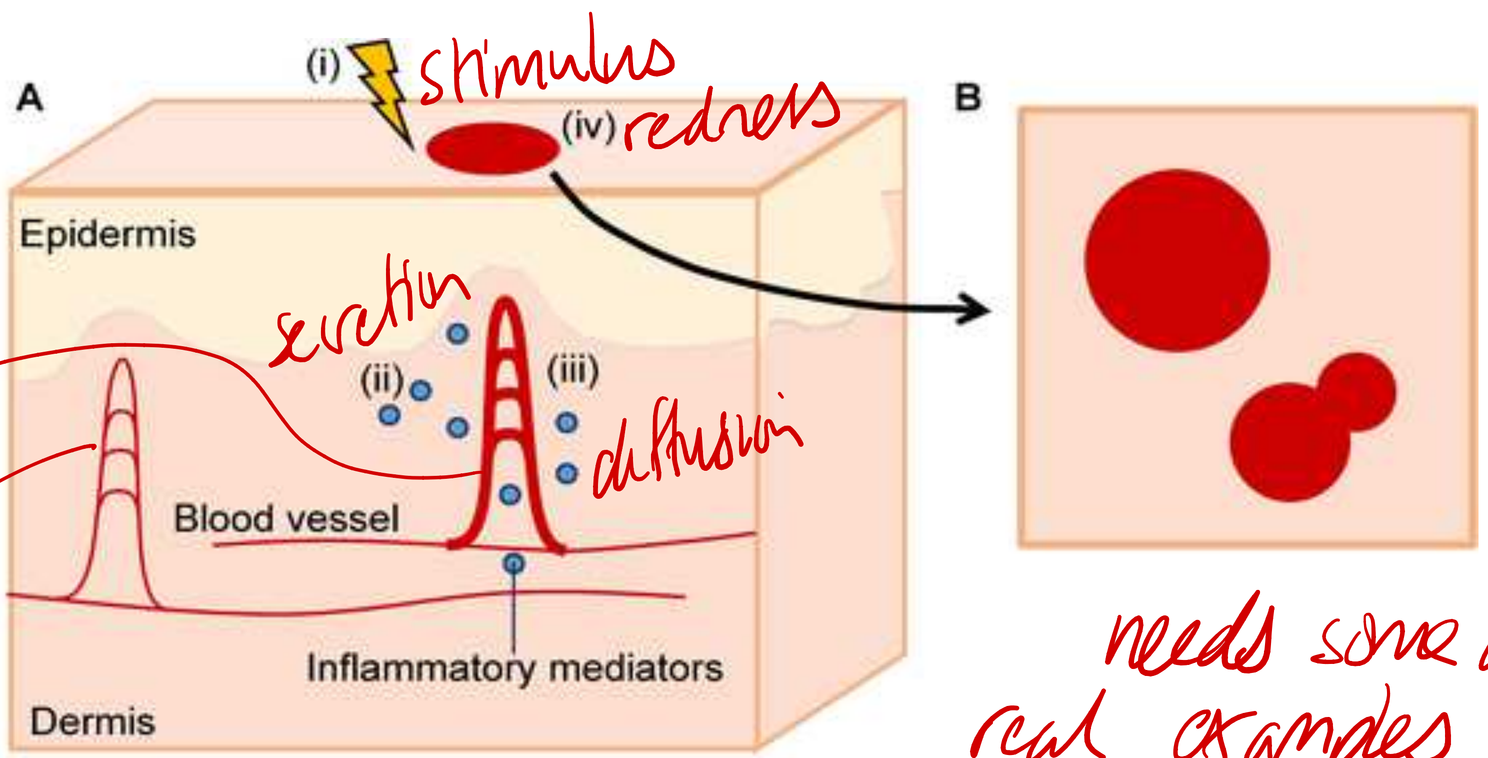

Dilated blood vessel (I thought these were sketches of a spatial distribution of inflammatory mediator)

needs some data/ real examples

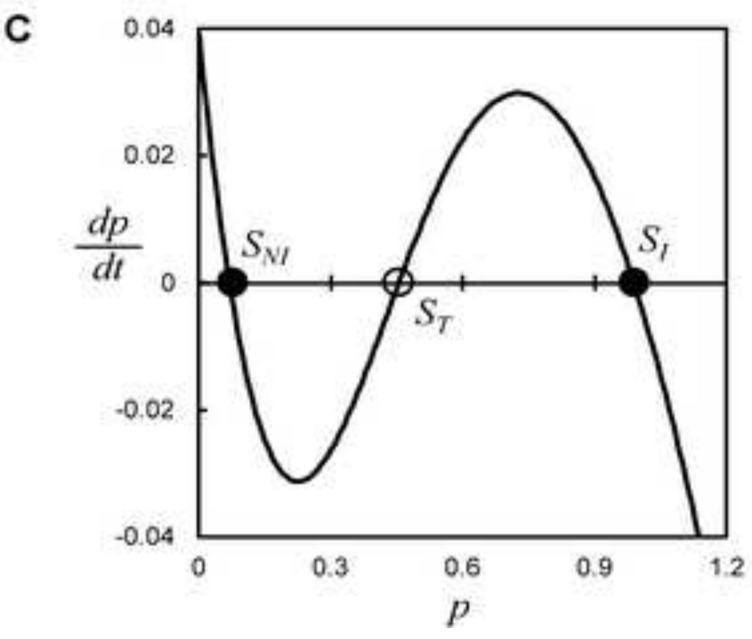

Fig 1. Modeling of the expansion of erythema.

(A) Process of the inflammatory response for erythema development. When keratinocytes in the epidermis and resident immune cells in the dermis are stimulated (i), they secrete inflammatory mediators that induce their own production from these mediator-secreting cells (ii). The mediators diffuse in the dermis and cause the dilation of local blood vessels (iii). The dilation appears as redness on the skin surface and erythema develops (iv).

(B) A two-dimensional illustration of erythema expansion showing sharply circumscribed boundaries and fusion.

(C) Kinetics of bistability in Eq. (1) represented by a production rate of mediators (dp/dt) as a function of the concentration (p). Two filled circles represent stable steady states (S_{NI}, S_I), whereas the hollow circle indicates an unstable steady state (S_T). This system can switch between the states of stable low (S_{NI}) and high (S_I) concentration states, depending on perturbation such as initial stimulation or diffused mediators ($\alpha = 1.0$).

Figure 2

Click here to access/download;Figure;Fig2.tif

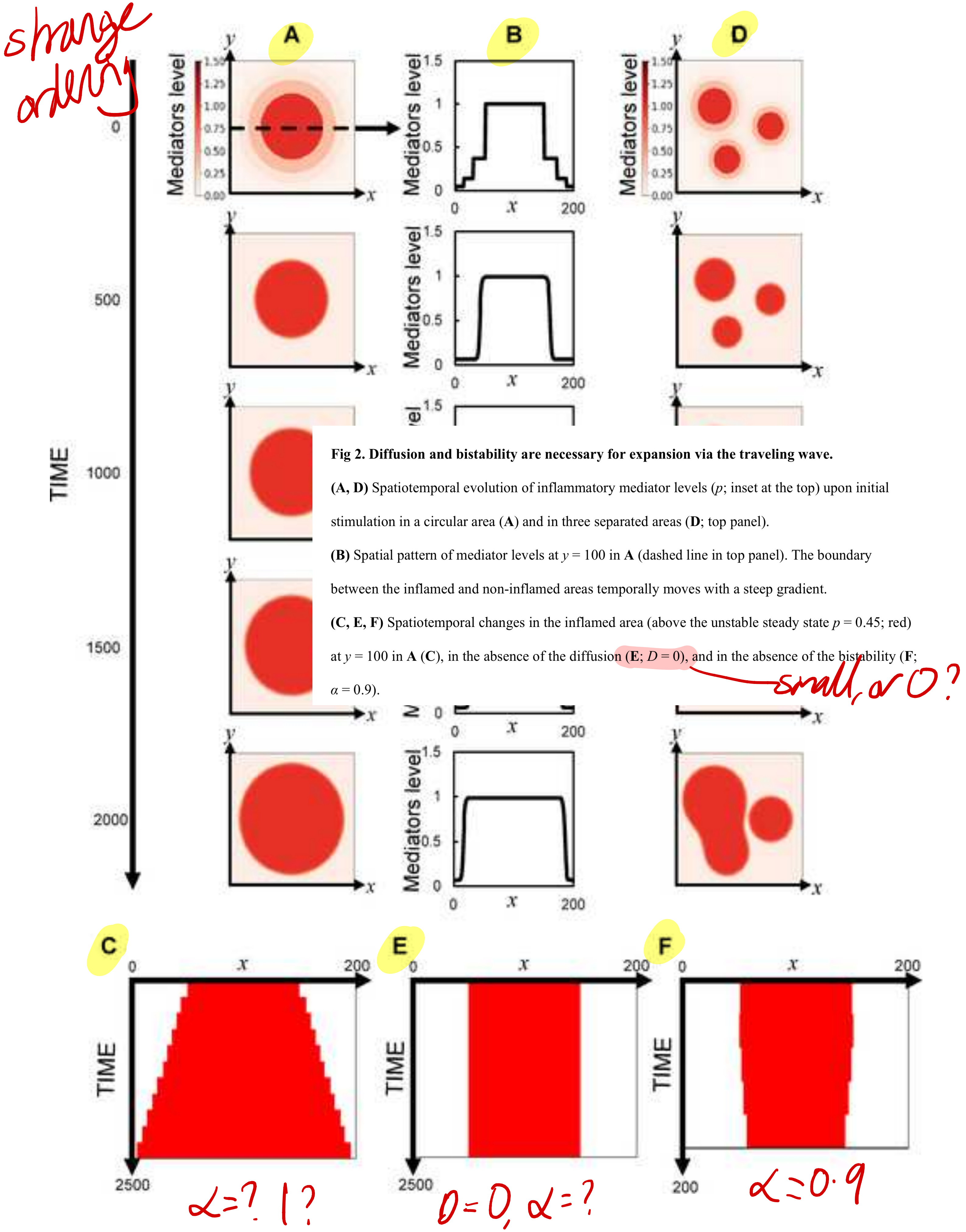

Fig 2. Diffusion and bistability are necessary for expansion via the traveling wave.

(A, D) Spatiotemporal evolution of inflammatory mediator levels (p; inset at the top) upon initial stimulation in a circular area (**A**) and in three separated areas (**D**; top panel).

(B) Spatial pattern of mediator levels at $y = 100$ in **A** (dashed line in top panel). The boundary between the inflamed and non-inflamed areas temporally moves with a steep gradient.

(C, E, F) Spatiotemporal changes in the inflamed area (above the unstable steady state $p = 0.45$; red) at $y = 100$ in **A** (**C**), in the absence of the diffusion (**E**; $D = 0$), and in the absence of the bistability (**F**; $\alpha = 0.9$).

Click here to access/download;Figure;Fig3.tif

Fig 3. Expansion speed controlled by diffusion and positive feedback for given bistability.

(A-D) The dependence of expansion speed on the diffusion coefficient (*D*; **A**), the production rate (*α*; **B**), the basal secretion rate (*β*; **C**), and the degradation rate (*γ*; **D**).

(E-G) Spatiotemporal changes of the inflamed area (above the unstable steady state *p* = 0.37; **E**, 0.42; **F**, 0.5; **G**, respectively) for three different values of production rate (E, F, G in **B**).

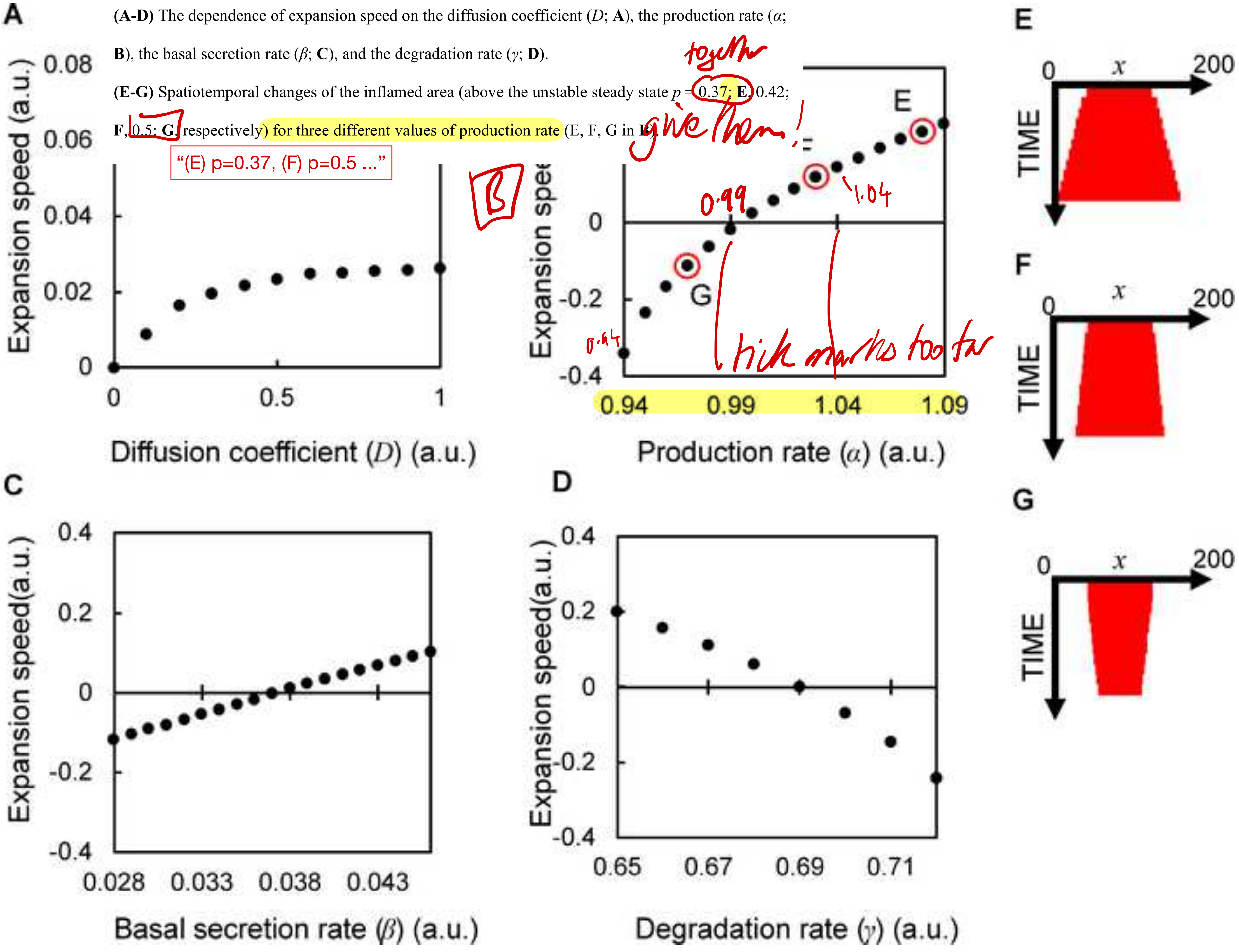

Figure 4

Click here to access/download;Figure;Fig4.tif

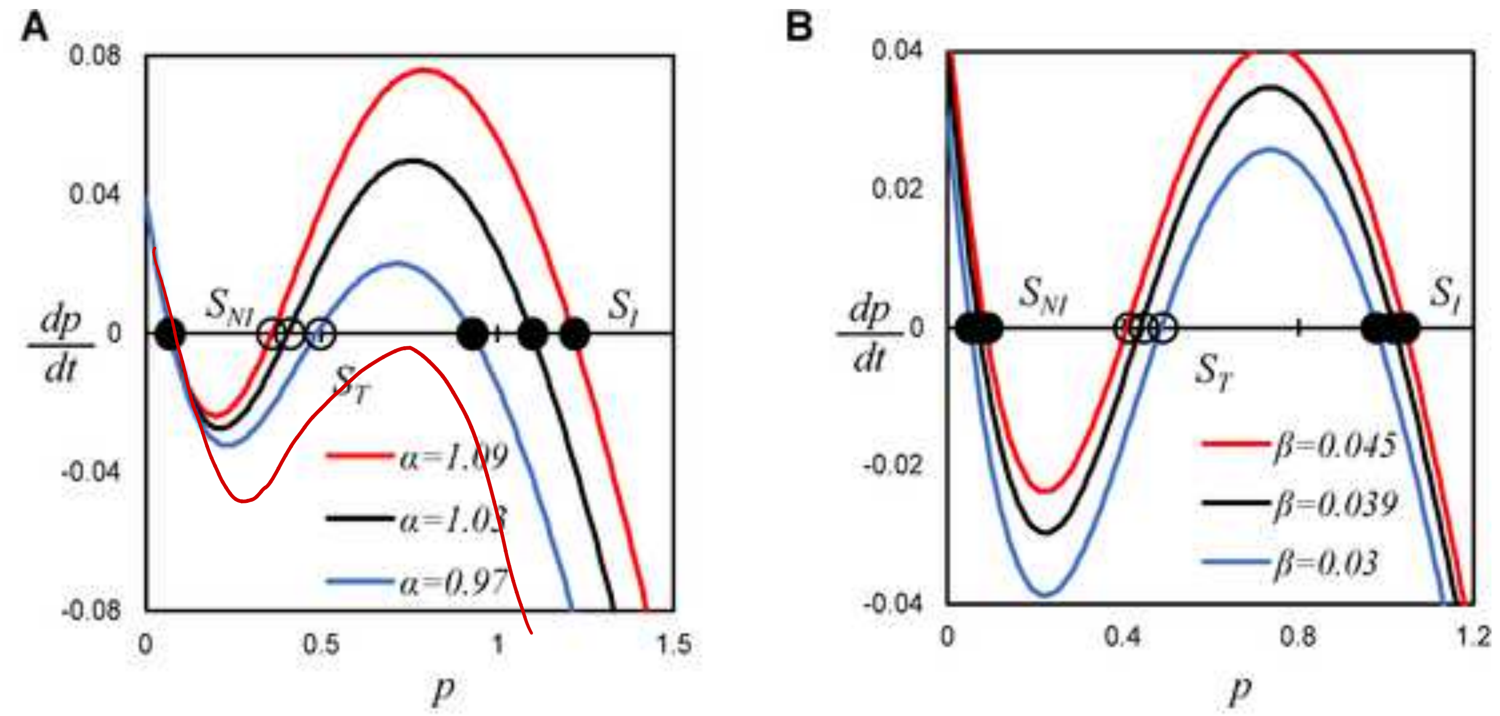

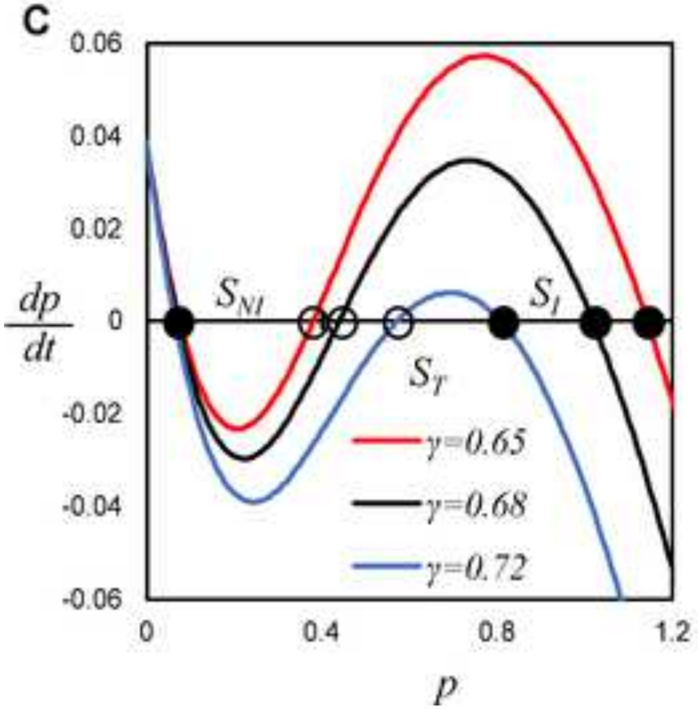

Fig 4. Balance of mediator concentration regulates expansion or shrinkage.

Kinetics of bistability in Eq. (1) represented by the production rate of mediators (*dp/dt*) as a function of the concentration (*p*). Two filled circles (S_{NI}, S_I) represent stable steady states, whereas the hollow circle (S_T) indicates an unstable steady state. (**A**) α = 1.09, 1.03, 0.97. (**B**) β = 0.045, 0.039, 0.03. (**C**) γ = 0.65, 0.68, 0.72. Blue and red lines represent models for erythema expansion and shrinkage, respectively, while the black line applies to zero expansion speed. cannot be true in PDE

other parameters are where?

Figure 5

Click here to access/download;Figure;Fig5.tif

Fig 5. Traveling wave of inflammatory response regulates the expansion or shrinkage of erythema.

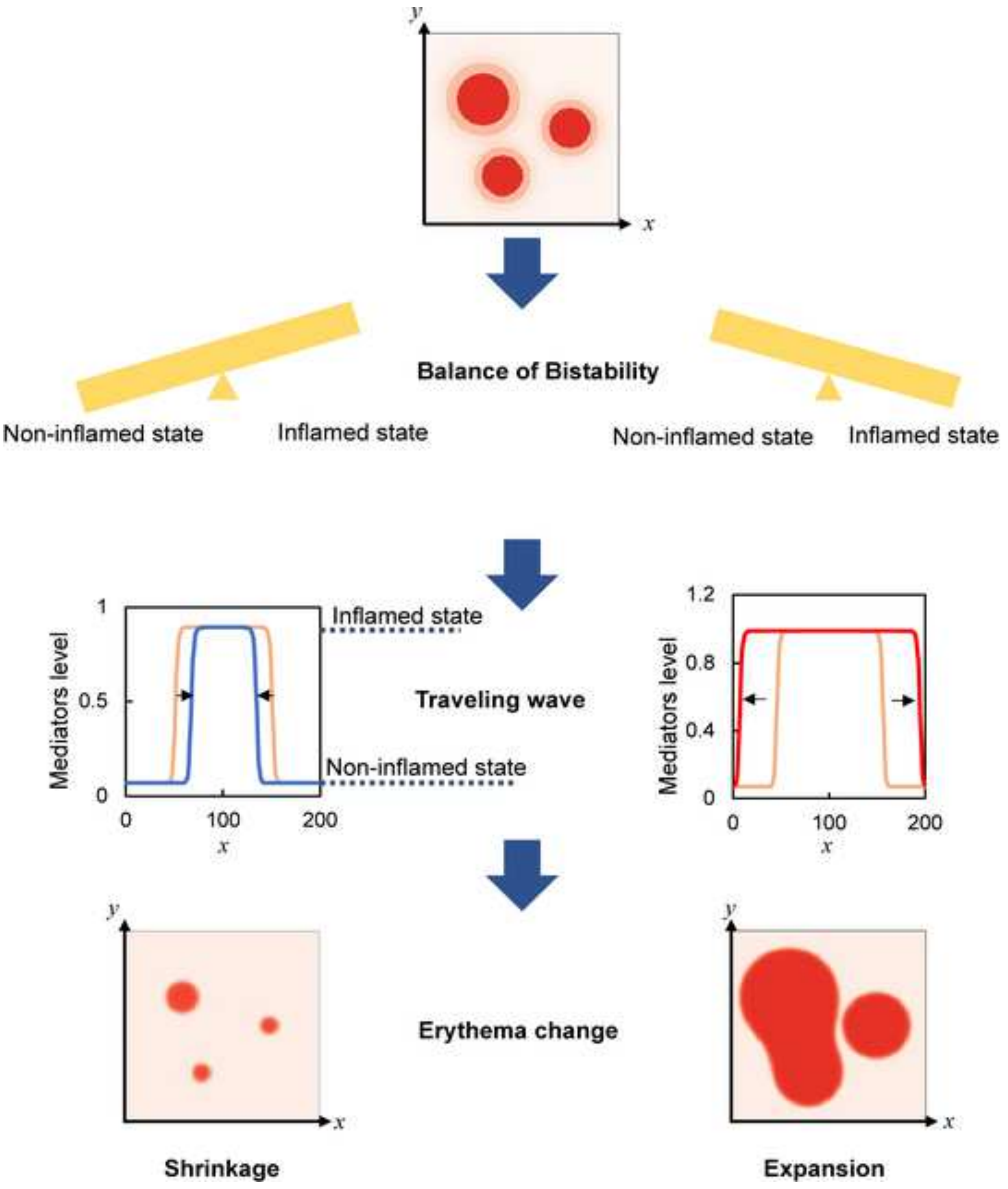

Click here to access/download
Supporting Information
Sudo_SupportingMaterials.pdf

Supporting Materials

Supporting Figures S1-S3

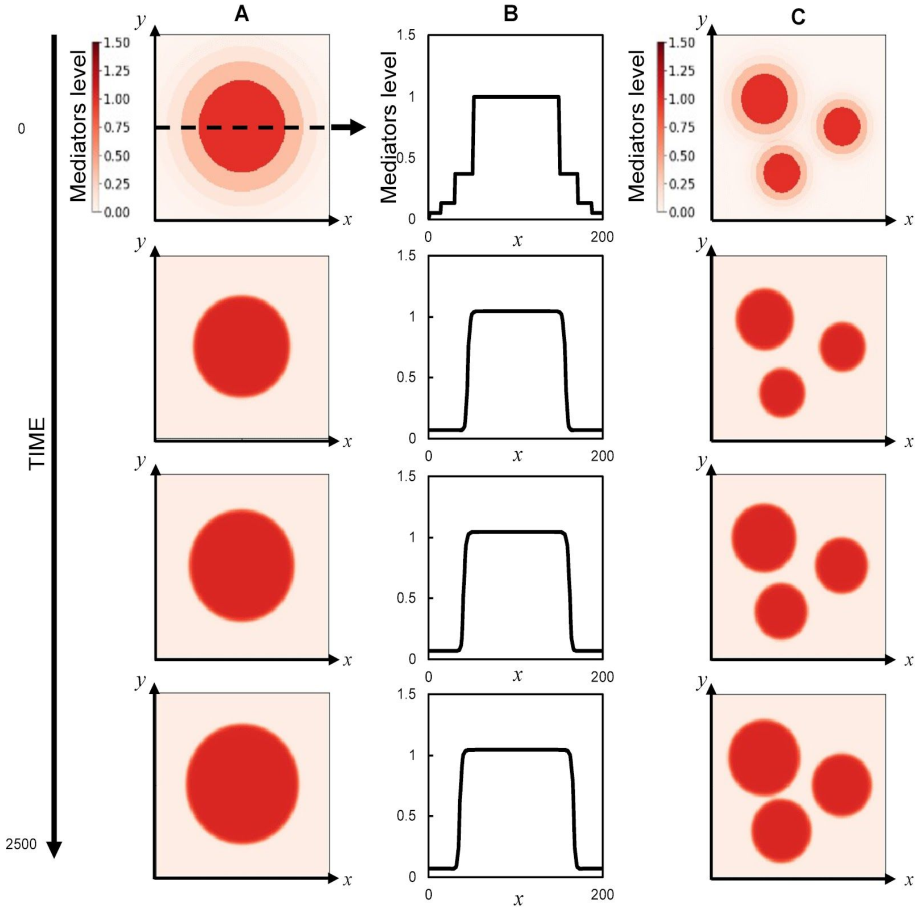

S1 Fig. Slow expansion.

(A, C) Spatiotemporal evolution of the inflammatory mediator levels (p, inset at the top) upon initial stimulation in a circular area (**A**) and in three separate areas (**C**, top panel). Parameter values are the same as those in Fig 3F.

(B) Spatial pattern of mediator levels at y = 100 in **A** (dashed line in top panel). The boundary between the inflamed and non-inflamed areas temporally moves with a steep gradient.

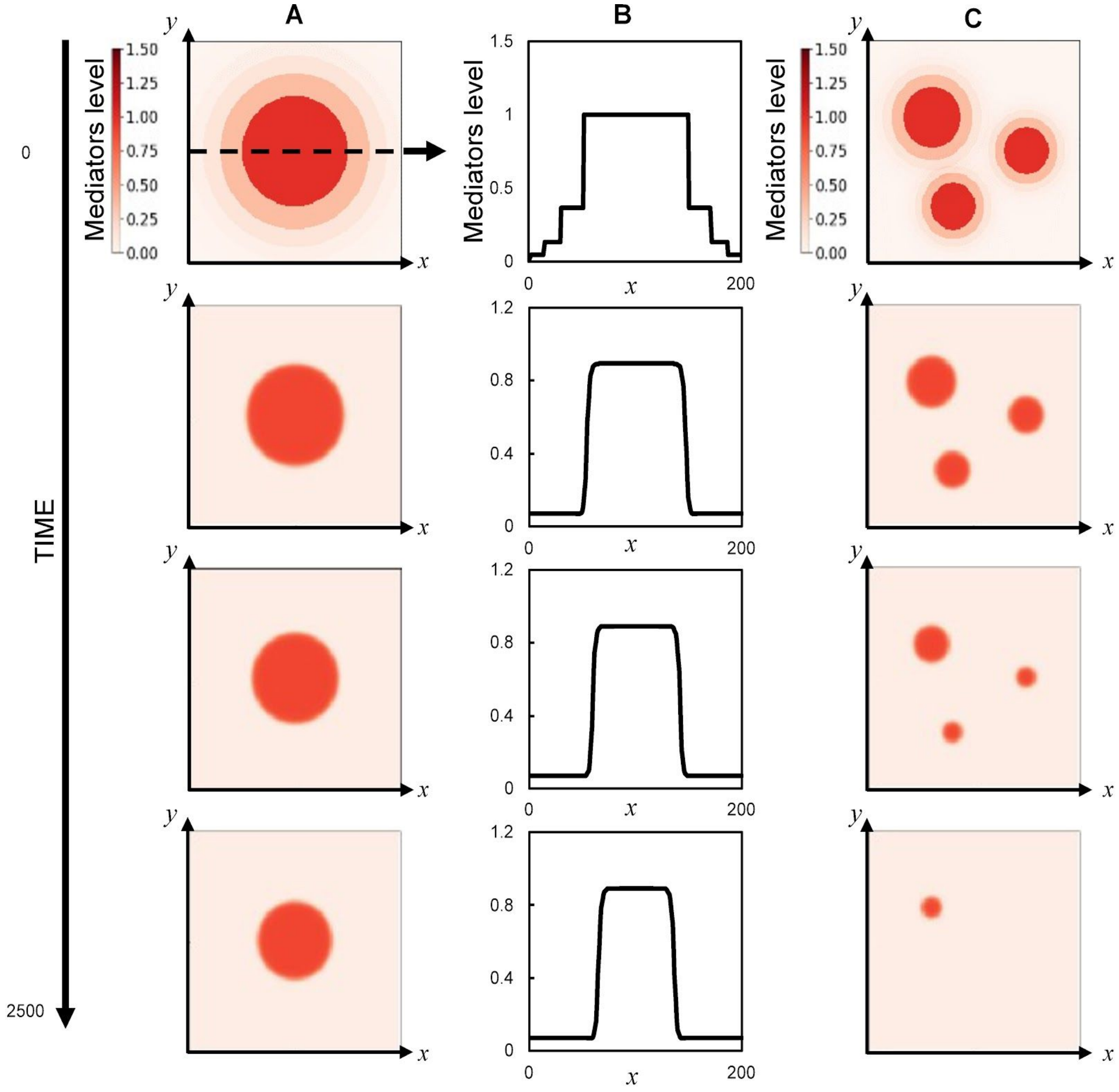

S2 Fig. Shrinkage.

(A, C) Spatiotemporal evolution of inflammatory mediator levels (*p*, inset at the top) upon initial stimulation in a circular area (**A**) and in three separated areas (**C**, top panel). Parameter values are the same as those in Fig 3G.

(B) Spatial pattern of mediator levels at y = 100 in **A** (dashed line in top panel). The boundary between the inflamed and non-inflamed areas temporally moves with a steep gradient.

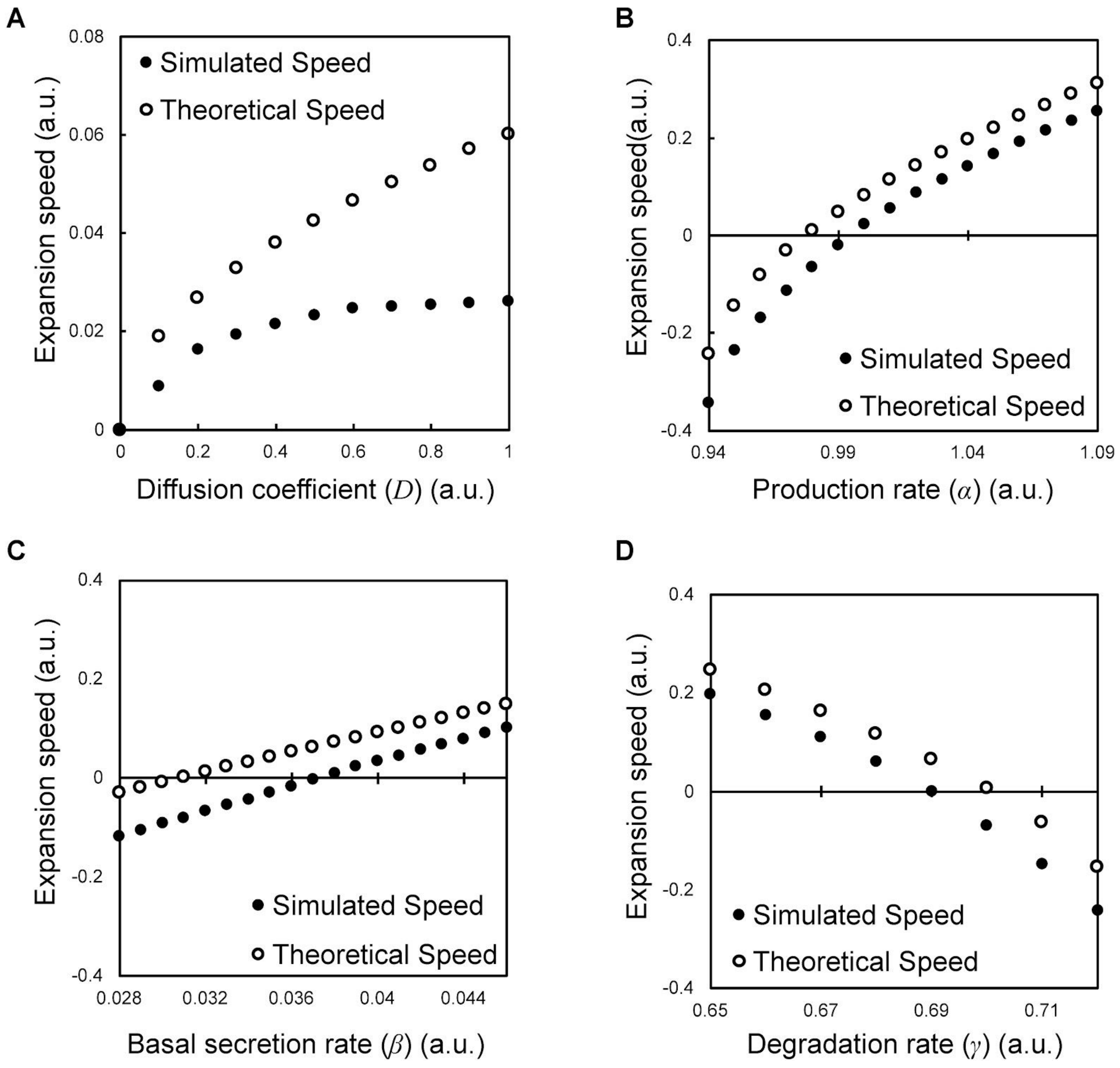

S3 Fig. Comparison between the simulated speed (Fig 3) and the speed calculated using Eq. (7).

Dependence of expansion speed on the diffusion coefficient (D, **A**), the production rate (α, **B**), the basal secretion rate (β, **C**), and the degradation rate (γ, **D**).

explain that one is for an approximation (the theoretical speed)
