# Supplementary material for: Traveling wave of inflammatory response to regulate the expansion or shrinkage of skin erythema
Source: PLoS One. 2022 Feb 9;17(2):e0263049. doi: 10.1371/journal.pone.0263049 (PMC8827459; doi:10.1371/journal.pone.0263049)
Supplement: Attachment — Submitted filename: Response to Reviwers (1).docx [file pone.0263049.s003.docx]

Response to Reviewer #1:

>Reviewer #1: I appreciate that the revision addressed some of the reviewers’ comments and improved the clarity of the manuscript to some extent. However, there are still a few scientific issues to be clarified, and too many issues with the lack of clarity in description for scientific papers. The science behind seems to be solid, but the paper writing is still poor, which is really unfortunate.

Authors: We thank you for providing your valuable comments on our manuscript. Following the reviewer’s suggestions, we have clarified model assumptions and results throughout the manuscript. In addition, the paper has been edited again by an experienced scientific editor, who has improved the grammar and stylistic expression of the paper.

>Line 14: what does “normal inflammatory response” mean?

Au: Our intended meaning of “normal inflammatory response” is an inflammatory response in healthy skin, which initiates a temporal increase in the level of mediators and returns to original levels. Following the reviewer's comment, we have added this explanation to the Background (p. 3 lines 46–48; revised manuscript).

>Line 23: “the circular inflamed area expands via the traveling wave from the noninflamed to the inflamed state” in the model simulation. But it cannot be claimed as is until it is confirmed experimentally.

Au: We have added the expression “a possible mechanism in which” to clarify that this sentence (p. 2 line 24; revised manuscript) refers to the model simulation.

>Line 26: “positive feedback becomes weak given the bistability” – why?

Au: To avoid such confusion, we have removed the expression “given the bistability” from the text (p. 2 line 27; revised manuscript).

>Line 28: This sentence does not make sense grammatically.

Au: We have revised the manuscript (p. 2 line 28–30; revised manuscript), and it has been proofread again by professional English language editor.

>Line 28: What does “approximately controlled” mean?

Au: In a precise mathematical sense, the velocity of the traveling wave depends on the steady state values and nonlinearity between the steady states in the model equation [Volpert V et al. 2009]. Here we approximated that the nonlinear term is a constant (*A* in Appendix) and theoretically derived the velocity of the traveling wave, which is proportional to the “balance” (Eq. 5). Even under this assumption, the theoretical velocity showed a similar dependence on the model parameters (i.e., a switch between expansion and shrinkage) to the velocity in the numerical simulations (S3 Fig). Thus, we conclude that balance is a major component controlling the velocity. Following the reviewer's comment, we have changed “approximately” to “mainly” in the text (p. 2 line 29; revised manuscript).

>Line 30: The wording “control principle” is an overstatement and should be removed from the manuscript. You cannot claim the result as a principle, unless you can demonstrate the general applicability of the “principle” to other systems. The science behind the reported work is solid, and it is sufficient to claim that the traveling wave could explain erythema expansion and shrinkage, without an overstatement which counteracts for the credibility of the paper.

Au: Thank you for your accurate evaluation of our study. We have reflected this comment by replacing the “control principle” with the “experimentally testable framework” (p. 2 line 31, p. 13 line 291, p. 16 line 342; revised manuscript).

>Line 75: I would rather say “can cause” and “can appear” unless you can verify it experimentally.

Au: We have reflected this by adding “can” as suggested (p. 4 line 75; revised manuscript).

>Line 106: non-dimensionalisation?

Au: Yes. We have used the word in the sentence (p. 5 line 105; revised manuscript) following your kind suggestion: “For this purpose, we non-dimensionalized Eq. (2) by normalizing the variables and parameters as follows (See Appendix for a detailed derivation):”

>Line 112: How does the parameter d affect? The figure only shows the case where d=0, but the choice of *d* also should have some effects on the existence of bistability.

Au: The parameter *d* affects the velocity of the traveling wave (Fig. 3A); however, it does not affect the existence of bistability. The parameter region exhibiting bistability in the absence of diffusion (*d* = 0) agreed with that for the traveling wave in the presence of diffusion (Fig. 3F).

>Line 121: Please clarify how you define “large”.

>Line 122: The relationship between models and the experimental/clinical observations is simply hypothetical. S_NI and S_I do not appear in the dermatology textbook cited there. Please make it clear that it is a speculation or a hypothesis.

Au: We have removed the expression “large” and revised this sentence (p. 6 line 121–123; revised manuscript) without referring to the “hypothetical” relationship as follows: “Finally, as an initial condition of the model simulation (Eq. 3), we referred to the physiological condition at the onset of erythema, where one or a few small (~1mm) inflamed areas exhibited a concentration of mediators above the threshold (*S_T_*) [1].”

>Line 124: Do you assume that the high *q* in the dermis appears as the erythema on the skin surface directly? It is a model assumption that needs to be articulated.

Au: Yes. Accordingly, we have articulated the assumption in the main text (p. 6 line 114–115; revised manuscript).

>Line 131: How was it “confirmed”? Please demonstrate the results.

Au: As requested, we have added the results to Fig. 2D and referred the figure (p. 7 line 133).

>Line 156: “whether diffusion…can cause”. This paper examined whether the model could demonstrate an expansion of the erythema as a result of diffusion and bistability. It needs to be clear that the results are about model simulation. Otherwise it would become an overstatement.

Au: We have added the expression “in the model” to clarify that this sentence (p. 8 line 160; revised manuscript) refers to the model simulation.

>Line 157: what does “consistently” mean? Always observed for different parameter values? What are the ranges investigated?

Au: Here, we intend to ensure that our simulation result is consistent with clinical observations of the erythema expansion. To avoid such confusion, we have revised the text (p. 8 line 161–163; revised manuscript) as follows: “The model simulations showed that a circular inflamed area was initially caused by a transient and local perturbation to the mediator’s concentration and subsequently expanded centrifugally over time (Fig. 2A), consistently with the expansion of erythema (Fig. 1B).”

>Line 160: How “large” is large?

Au: We removed the expression “large” from the text (p. 8 line 163–165; revised manuscript) and revised the text as follows: “During the expansion, the inflamed area maintained a steep gradient of concentration at the boundary (Fig. 2B) and increased the diameter at a constant rate (velocity) over time (Figs. 2C and D).”

>Line 169: please clarify why you always consider the case where d=0.

Au: Here, we aimed to examine whether diffusion is necessary for the expansion of the inflamed area in our simulation. To clarify this, we now revised the text (p. 8 line 171–172; revised manuscript) as follows: “Without diffusion (i.e., *d* = 0), an inflamed area appeared; however, this area did not expand and remained constant over time (S1Fig. C).”

>Line 181: Irreversibility of the transition does not seem to have been mentioned before.

Au: We have replaced the term “irreversible” with “selective” throughout the paper (p. 9 line 184 and line 190; revised manuscript).

>Line 248: “smaller” and “larger” than what?

Au: Following your comments, we restructured the sentence without using “larger” nor “smaller” (p. 12 line 251–253; revised manuscript) as follows: “When *S_T_* is at an equal distance from *S_NI_* and *S_I_*, given a decrease in the maximum production rate (*a*), the velocity is suppressed toward zero (Figs 4A and C).”

>Line 272: What does “persistently” mean?

Au: Our intended meaning of “persistently” is that in pathological skin showing the erythema expansion, the inflammatory response persists, and the concentration of mediators fails to return to the original level. We have revised these sentences (p. 13 line 274–276; revised manuscript) to clarify the statement.

>Line 295: Figs 2F-J show only the cases for circumscribed erythema and do not correspond to the description of a poorly circumscribed lesion.

Au: Thank you for pointing out this important issue. We have summarized definitions of well- and poorly-circumscribed erythema and discussed the correspondence with our model results in a subsection titled “Expansion of a well- and poorly-circumscribed erythema” in the Discussion (p. 14 line 292–309; revised manuscript).

>Line 300: Section on “Potential application of treatments” is a quite of stretch and speculative, given the theoretical analysis conducted in this paper. It may be worth removing to make the manuscript scientifically more solid.

Au: We have removed the speculative sentences highlighted by the reviewer, and reorganized the subsection (p. 14–15 line 310–323; revised manuscript), focusing on the relevance of our mathematical model to the biological factors of the skin.

>Line 304: skin microbiome composition also affects the skin barrier integrity, and application of moisturizers also affects the skin microbiome. We cannot separate the effects of the skin microbiome and moisturizers.

Au: We have revised the paragraph (p. 14–15 line 310–323; revised manuscript) and deleted reference [33] to focus on the probiotics without referring to the moisturizers, following the reviewer’s suggestion.

Minor comment

>- I would suggest reducing the use of “this” in the manuscript. It is not always clear what “this” designates for.

Au: We have replaced “this” with clearer terms throughout the paper.

>- Could authors share the code on GitHub?

Au: Yes. We have shared the code on GitHub, and have described the URL in main text (p. 7 line 135–136):

<https://github.com/MakiSudo/Travelingwave_Simulation/blob/bc2c10ddd5eff8db374b0804e11a63ef3c0e766a/Simulationcode.c>

Response to Reviewer #2

>Reviewer #2: I am satisfied with the revised version. No further comments.

Au: We appreciate your positive evaluation and many insightful comments on our manuscript.
